# The effect of pre-endoscopy maltodextrin beverage on gastric residual volume and patient’s well-being: a randomised controlled trial

**DOI:** 10.1038/s41598-023-47357-5

**Published:** 2023-11-16

**Authors:** Mohd Firdaus Zulkifli, Mohd Nizam Md Hashim, Zalina Zahari, Michael Pak-Kai Wong, Syed Hassan Syed Abd Aziz, Maya Mazuwin Yahya, Wan Zainira Wan Zain, Andee Dzulkarnaen Zakaria, Rosnelifaizur Ramely, Soh Jien Yen, Muhammad Faeid Othman

**Affiliations:** 1 Department of Surgery, School of Medical Sciences, Universiti Sains Malaysia (USM), 16150 Kubang Kerian, Kelantan Malaysia; 2 Hospital Universiti Sains Malaysia, Kubang Kerian, Kelantan Malaysia; 3 Faculty of Pharmacy, Universiti Sultan Zainal Abidin (UniSZA), Besut Campus, 22200 Besut, Terengganu Malaysia; 4 Endoscopy Unit, Hospital Universiti Sains Malaysia, Kubang Kerian, Kelantan Malaysia

**Keywords:** Randomized controlled trials, Oesophagogastroscopy

## Abstract

Prolonged fasting prior to oesophagogastroduodenoscopy (OGDS) could be noxious to patients’ well-being. Strict fasting protocol has been used prior to OGDS with the concern of reduced visibility or suboptimal endoscopic assessment. Maltodextrin beverages were also commonly used as the pre-operative carbohydrate loading in enhanced recovery after surgery (ERAS) protocol. Our study aimed to look for the effects of maltodextrin beverage 2 h before OGDS on gastric residual volume and patient’s well-being scores. This was a single-blinded, stratified randomised controlled trial, comparing control group (A, received 400 ml of plain water) and carbohydrate loading group (B, received 400 ml of Carborie). The primary objectives were to measure the gastric residual volume (GRV) and patient’s well-being scores using visual analogue scale (VAS) scores for hunger, thirst, anxiety, tiredness and general discomfort. Of 80 randomised patients, 78 completed the study (38 received plain water and 40 Carborie). The median (IQR) GRV was not significantly different between group A and B (5.0 ml (20) vs 4.0 ml (19), *p* = 0.777). Both groups showed significant reduction in VAS scores in all five parameters (*p* ≤ 0.001). There were no complications attributed to endoscopy in either group. Pre-endoscopy maltodextrin beverage is as safe as clear water with improved patient’s well-being in both groups.

**Clinical Trial Registration**: NCT05106933.

## Introduction

Upper gastrointestinal (GI) endoscopy or oesophagogastroduodenoscopy (OGDS) is a procedure widely used in surgical and medical practice to examine the lining of the upper part of the GI tract using the thin, flexible tube that has its lens, light source and will view the images on a monitor. It has diagnostic and therapeutic capabilities for any pathologies involving the upper GI tract.

The European Society of Gastrointestinal Endoscopy (ESGE) and the United European Gastroenterology (UEG) have stated the quality of endoscopic outcome as a major priority and reported the multistep process for the methodology to develop performance measures during upper GI endoscopy^1^. The ESGE has described 6 keys and 5 minor performance measures as tools to improve the quality of endoscopy and patient outcomes. One of the key performance measures includes fasting instruction before upper endoscopy. Upper endoscopy is conventionally practiced under moderate sedation and after a fasting period of at least 6 h^2^. Many local institutes still practising nil by mouth from midnight that usually extends the fasting period for more than 8 h. However, prolonged fasting period might lead to patient discomfort, anxiety and thirst^3^.

There is no standard practice for pre-procedural fasting that has been universally accepted and many literatures differ in the recommendation for oral intake before the procedure that requires sedation. The American Society of Anesthesiologists (ASA) guideline has recommended 2-h fasting for clear fluid and 6-h fasting for solid food before procedural sedation^4^. For upper endoscopy, at least two studies for the last decade were found addressed this duration of fasting before elective upper endoscopy^5, 6^. The ESGE guideline in their performance measures has suggested for 2 h of water intake before the scheduled upper endoscopy. Previous reports have shown that clear fluid containing carbohydrate and electrolytes 2 to 4 h before the elective procedure is safe and does not interfere with gastric pH and gastric residual volume (GRV) when compared to longer fasting period^7, 8^. The carbohydrate-rich whey protein beverage was shown to yield higher GRV compared to clear water but improved patient’s well-being^9^.

In this study, we compared the GRV and patient’s well-being score of those participants who were served maltodextrin only beverage and plain water 2 h before gastroscopy.

## Methods

### Study design

This was a single-center, stratified (diabetes mellitus (DM) and non-diabetes mellitus) with gender balanced randomisation (1:1), single-blinded, placebo-controlled, parallel-group study regarding the effect of pre-endoscopy beverage served at 2 h prior to the outpatient upper endoscopic procedure. The endoscopists that performed this procedure and the staff nurse assessing the patient’s well-being both blinded.

The study protocol conforms to the ethical guidelines of the 1975 Declaration of Helsinki (6th revision, 2008) as reflected in a priori approval by the Human Research Ethics Committee, Universiti Sains Malaysia in Kelantan, Malaysia (USM/JEPeM/20080414). This study was registered at ClinicalTrials.gov (NCT05106933).

### Participants

Patients who were scheduled for elective gastroscopy from January to August 2021 were invited to participate voluntarily in this study. Patients aged more than 18 years old with symptoms of dyspepsia and agreed to participate in the study were included. Those with history of upper gastrointestinal surgery, clinically unstable condition and with active upper GI bleeding were excluded from the study. Pregnant lady and patients who were on nasogastric feeding tube were also not eligible for this study as it may interrupt the measurement of GRV. This study was conducted in the Endoscopy Unit, Hospital Universiti Sains Malaysia, Kubang Kerian, Malaysia.

A total of 80 patients who fulfilled the inclusion criteria were selected for randomisation process. Written informed consent was obtained before randomization.

### Intervention

For allocation of the participants, a computer-generated list of random numbers was used, which was prepared by an investigator with no clinical involvement in the study. Randomisation sequence was created using https://www.sealedenvelope.com/simple-randomiser/v1/lists and was stratified by patients who having DM and non-DM with a 1:1 allocation using random block size of 6.

The allocation sequence was concealed from researcher enrolling and assessing participants in sequentially numbered, opaque, sealed and stapled envelopes. To prevent subversion of the allocation sequence, the name and identification number of the participant was written on a book together with the serial number on the envelope. The details in the book were kept confidentially. Only after the enrolled participants completed all baseline assessments, the corresponding envelope was opened by the trained staff who involved in preparing the drink. The staff need to ensure that the envelop was still sealed when receiving it. The staff prepared the drink into an identical container according to randomisation.

Participants were randomised into control group (group A, 400 ml plain water) and maltodextrin beverage group (group B, 400 ml of Carborie, 50 g of carbohydrate, protein-free) (Valens Nutrition, Kuala Lumpur, Malaysia). All the participants were asked to fast from midnight, more than 8 h prior to examination. Participants consumed the drink over 10 min. Participants were not allowed to leave the endoscopy room until their procedure were completed to prevent consumption of other drinks or foods. Gastroscopy was performed after 2 h of beverage consumption, following the standard protocol. The endoscopist was advice to use only air inflation and not to use any water flushing; only lens flushing was allowed to ensure clear visualization of the gastric content. The visualized pooling of fluid within the stomach was then aspirated via direct visualisation with the endoscope. The aspirated fluid was collected in the suction reservoir to be recorded as GRV.

Participant’s well-being score (consisted of 5 parameters included hunger, thirst, anxiety, tiredness and general discomfort) was assessed using visual analogue scale (VAS). Each scale consisted of ungraded, horizontal lines anchored at two ends. The left end of the scale represented “not at all” which score: 0 mm and the right end represented and “the most imaginable” which score: 100 mm. The trained staff nurse asked the participant regarding the level of 5 parameters and the participant marked an [X] somewhere along the horizontal line to complete the scale before administration of the drink and before OGDS procedure.

### Outcomes

The primary objective was to compare GRV (ml) between participant in plain water group (group A) and carbohydrate loading group (group B). The aspirated fluid was collected in the suction reservoir and GRV was measured. The second objective was to compare patient’s well-being score for group A and B. Participant’s well-being score was assessed using VAS which consisted of 5 parameters: hunger, thirst, anxiety, tiredness and general discomfort.

### Statistical analysis

The numerical data were presented as mean and standard deviation (SD) or median and interquartile range (IQR) for continuous data depending on their normality. For categorical data, frequency and percentage were used. Primarily, the normality of data distribution was evaluated by the box plot, graph and test of normality for both groups. To compare GRV between the two groups, the Mann–Whitney U test was performed. The Wilcoxon signed-rank test was used to compare participants’ well-being scores before and after administration of the drink in each group. The Mann–Whitney U test was conducted to compare patient’s well-being score between group A and B before and after administration of the drink. Data analyses were performed using IBM SPSS Statistics for Windows, Version 27.0 (IBM Corp., Armonk, NY, USA). The limit of significance was set at 0.05.

### Sample size estimation

The sample size was calculated using two means formula using http://www.openepi.com/SampleSize/SSMean.html^10^ for the first objective and paired difference formula using http://statulator.com/SampleSize/ss2PM.html for the second objective.

For the first objective, which is to compare GRV between the two groups, the ratio of sample size (group A/group B) was set as 1. The sample size was calculated using the standard deviation (18.46 ml) and difference between the means (12.5 ml). Based on these two estimates, the calculated sample size for this objective was 35 subjects per group.

For the second objective, which is to determine patient’s well-being for group A and B, the expected standard deviation of the paired differences^14^ was set as 2 times the expected mean of the paired differences^7^. The calculated sample size for the second objective was 34 subjects per group. The final calculated sample size was 39 subjects per group (with two-sided 5% significant level and a power of 80%, in view of dropout rate of 10%).

### Ethics approval and consent to participate

An ethical clearance was obtained from the Human Research Ethics Committee, Universiti Sains Malaysia in Kelantan, Malaysia (Institutional Review Board registration number: USM/JEPeM/20080414). All procedures followed were in accordance with the ethical standards of the responsible committee on human experimentation and with the 1975 Declaration of Helsinki (6th revision, 2008).

## Results

A total of 457 patients were referred for elective gastroscopy during the study period from 1st January until 31st August 2021. However, a total of 377 patients were excluded from this study for the following reasons; decline to participate in the study, patients who concurrently underwent gastroscopy and colonoscopy, pregnant patients, and those on nasogastric tube.

**Figure 1:**
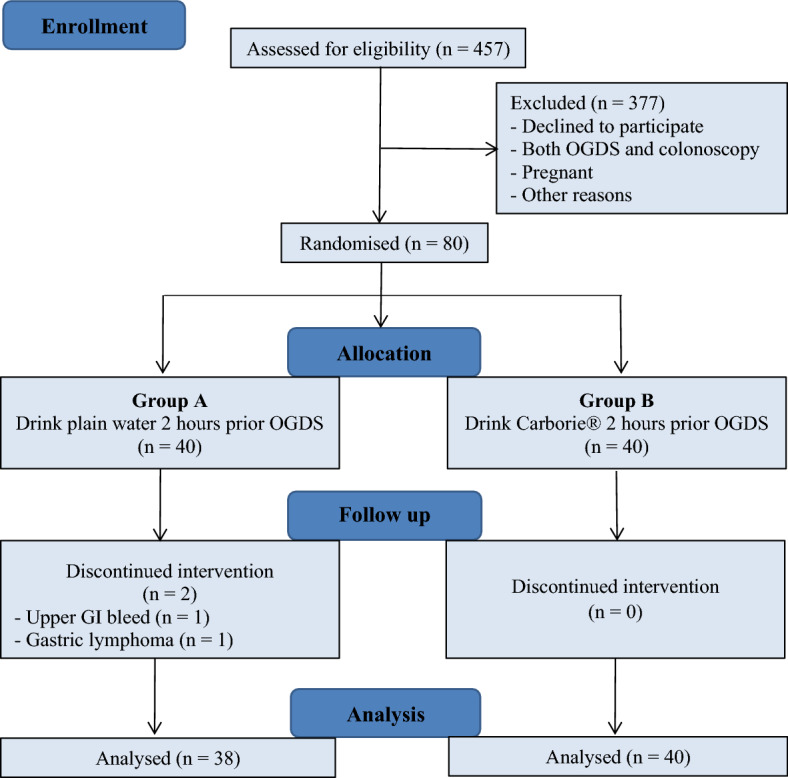
Consort flow chart of the study.

After randomisation, the 80 patients were allocated into 2 groups; 40 patients were given 400 ml plain water (Group A, control group) and 40 patients were given 400 ml Carborie (Group B, intervention group) 2 h prior to gastroscopy. Two patients in group A did not complete the study due to active upper GI bleeding during the procedure and gastric malignancy (Fig. 1).

The sociodemography data of the 78 patients were presented in Table 1; no significant differences existed between the 2 groups in terms of age, gender, body mass index (BMI) and DM status. Indications for gastroscopy were those age more than 18 years old with symptoms of dyspepsia.

**Table 1:** Demographic data of the two groups.

	Group A (n = 38)	Group B (n = 40)	*p* value*
Age, years, mean (SD)	48.0 (32)	42.5 (27)	0.745
Gender, n (%)			
Male	19 (52.8)	17 (47.2)	0.507
Female	19 (45.2)	23 (54.8)	
BMI, kg/m^2^, mean (SD)	27.6 (9.77)	26.6 (7.13)	0.254
DM status, n (%)			
Non-DM	25 (46.3)	29 (53.7)	0.521
DM	13 (54.2)	11 (45.8)	

Group A = patients were given 400 ml plain water (control group) 2 h prior to the esophagogastroduodenoscopy (OGDS); Group B = patients were given 400 ml Carborie® (carbohydrate loading) 2 h prior to the esophagogastroduodenoscopy (OGDS).

*SD* standard deviation, *BMI* body mass index, *DM* diabetes mellitus.

*p values were calculated using the Student’s *t*-test for continuous variables and Chi-square test for categorical variables.

### Gastric residual volume (GRV)

The median (IQR) GRV was not significantly different between group A and B (5.0 ml (20) vs 4.0 ml (19), p = 0.777). According to the Ministry of Health Malaysia, obesity in adults is defined as BMI greater than 27.5 kg/m^2^. In group A, non-obese patients (n = 20) had higher GRV compared to obese patients (n = 18) (median (IQR) = 10 ml (18) vs 0 ml (21)). Conversely, in group B, non-obese patients (n = 25) had lower GRV compared to obese patients (n = 15) (median (IQR) = 2 ml (12) vs 10 ml (30)). However, the differences were not statistically significant (*p* = 0.109 and 0.111, respectively) (Table 2).

**Table 2:** Gastric residual volume (GRV) of the two groups.

	Group A (n = 38)	Group B (n = 40)	*p* value*
GRV, ml, median (IQR)	5.0 (20)	4.0 (19)	0.777
Obesity			
Non-obese, BMI ≤ 27.5 kg/m^2^	10.0 (18)^#^	2.0 (12)^##^	0.109^#^
Obese, BMI > 27.5 kg/m^2^	0.0 (21)^#^	10.0 (30)^##^	0.111^##^
DM status			
Non-DM	2.0 (18)^###^	2.0 (20)^####^	0.162^###^
DM	10.0 (34)^###^	10.0 (13)^####^	0.255^####^

Group A = patients were given 400 ml plain water (control group) 2 h prior to the esophagogastroduodenoscopy (OGDS); Group B = patients were given 400 ml Carborie® (carbohydrate loading) 2 h prior to the esophagogastroduodenoscopy (OGDS).

*GRV* gastric residual volume, *IQR* interquartile range, *SD* standard deviation, *BMI* body mass index, *DM* diabetes mellitus.

*p values were calculated using the Mann–Whitney U test.

^#^*p* value was calculated for gastric residual volume in group A between non-obese (n = 20) and obese patients (n = 18).

^##^*p* value was calculated for gastric residual volume in group B between non-obese (n = 25) and obese patients (n = 15).

^###^*p* value was calculated for gastric residual volume in group A between patients without diabetes mellitus (n = 25) and with diabetes mellitus (n = 13).

^####^*p* value was calculated for gastric residual volume in group B between patients without diabetes mellitus (n = 29) and with diabetes mellitus (n = 11).

For patients with underlying DM, GRV was higher in both groups. In group A, patients without DM (n = 25) had lower GRV compared to patients with DM (n = 13) (median (IQR) = 2 ml (18) vs 10 ml (34)). Similarly, in group B, patients without DM (n = 29) had lower GRV compared to patients with DM (n = 11) (median (IQR) = 2 ml (20) vs 10 ml (13)). However, the differences were not statistically significant (*p* = 0.162 and 0.255, respectively) (Table 2).

The categorical GRV using cut-off of 20 ml was analysed in group A and B. However, no statistically significant difference was found between patient without DM and with DM (*p* = 0.289 and 0.859, respectively). Only two (18.2%) patients with DM in group B had GRV more than 20 ml (Supplementary Table S1).

We found a statistically significant difference in categorical GRV between non-obese and obese patients in group B (*p* = 0.014). There were six (40.0%) obese patients with DM in group B who had GRV more than 20 ml (Supplementary Table S2). Out of this, two patients were morbidly obese who were scheduled for gastroscopy assessments before bariatric surgery. About 40 ml GRV was aspirated out in both patients. Similarly, in group A, we found two patients were morbidly obese with GRV more than 20 ml. However, no statistically significant difference in categorical GRV between non-obese and obese patients in group A (*p* = 0.867).

### Patient’s well-being scores

Patient’s well-being score (hunger, thirst, anxiety, tiredness and general discomfort) was assessed using VAS. Patients’ well-being scores before and after administration of the drink in each group showed significant reduction in VAS scores in all five parameters (*p* ≤ 0.001) (Table 3).

**Table 3:** Patient’s well-being score in the two groups before and after administration of the drink.

Patient’s well-being score	Group	Pre-intervention	Post-intervention	*p* value*
Hunger, median (IQR)	A	27.3 (28.5)	10.5 (21.1)	< 0.001
	B	37.5 (34.5)	17.0 (27.5)	< 0.001
	*p* value**	0.168	0.293	
Thirst, median (IQR)	A	29.0 (39.3)	6.0 (16.8)	< 0.001
	B	39.0 (31.3)	6.5 (20.6)	< 0.001
	*p* value**	0.201	0.756	
Anxiety, median (IQR)	A	30.0 (33.3)	18.0 (28.5)	0.001
	B	43.0 (36.0)	22.3 (33.8)	< 0.001
	*p* value**	0.019	0.213	
Tiredness, median (IQR)	A	19.5 (28.8)	9.0 (19.5)	< 0.001
	B	25.0 (34.0)	16.5 (28.3)	< 0.001
	*p* value**	0.187	0.106	
General discomfort, median (IQR)	A	20.0 (26.3)	10.5 (19.5)	< 0.001
	B	20.0 (36.8)	10.5 (18.5)	< 0.001
	*p* value**	0.649	0.841	

*The Wilcoxon signed-rank test was used to compare the participants’ well-being scores before and after administration of the drink within each group.

**The Mann–Whitney U test was used to compare participants’ well-being scores before and after administration of the drink between group A and group B.

Patients’ well-being score between group A and B before and after administration of the drink were not statistically significant difference. However, the anxiety scores before administration of drink were found to be higher in group B (median (IQR): 30.0 (33.3) vs 43.0 (36.0), *p* = 0.019) (Table 3).

## Discussion

Patients who underwent upper endoscopy are at risk to develop gastric acid aspiration into the lung because they are unable to protect their airway as endoscopic affects the glottis closure. The risk of pulmonary aspiration is higher in patient with gastric fluid greater than 0.3 ml/kg body weight (20–25 ml in adult) ml and gastric pH less than 2.5^11–13^. It has become a standard practice for most of the unit for routine order nil by mouth from midnight that usually extend for a longer period. The reason for this is to prevent pulmonary aspiration and allowing complete endoscopic assessment of the gastric mucosa. However, this practice has changed with introduction of enhanced recovery after surgery (ERAS) protocol. ERAS program initiated by Professor Henrik Kehlet in 1990s, aimed to modify body response to major surgery physiologically and psychologically^14, 15^. Pre-operative carbohydrate loading up to 2 h prior to operation and avoidance of prolong fasting are the important steps in ERAS protocol^15^.

This study aimed to compare the GRV and patient’s well-being score in patients who received carbohydrate-rich beverage with plain water 2 h before gastroscopy. There was no significant difference in GRV between patients who received carbohydrate loading and plain water (4 ml and 5 ml, respectively). The European Society of Gastrointestinal Endoscopy (ESGE) guideline has recommended that patient should be fasting at least six hours of solid food and 2 h of clear fluid before the procedure for safety and complete endoscopic assessment^1^.

The acceptance of universal “fasting guidelines” before elective surgery plays an important role in mitigating risk by limiting residual gastric contents during the immediate pre-operative period. However, these guidelines cannot be applied in patients with particular physiologic states such as pregnancy, those undergoing emergency surgery or those with coexisting medical conditions^16^.

In our study, GRV measurement method was more accurate as it was direct aspiration and visualisation using gastroscopy. In the past study designs, measurement of GRV was made using indirect measurements either by aspiration from nasogastric tube or ultrasound assessment^12, 17, 18^. Hence, there is possibility of over or under estimation of residual gastric volume.

Obese patients were thought to have higher volume of gastric reservoir content compare to normal-weight patients due to delay gastric emptying. Thus, obese surgical patients have been presumed an increased risk of pulmonary aspiration syndrome. The available evidences on relationship of BMI and GRV were heterogenous^19, 20^. A prospective observational study showed that no significant difference of GRV assessment by gastric ultrasound in fasting condition between obese and normal-weight patients^18^. Gastric emptying rate also was found to be similar between obese and non-obese patients^21^. From our data, we created categorical GRV of 20 ml as a cut-off value so that data could be normally distributed. When using cut-off value of 20 ml, there were 6 (40%) obese patients those who received maltodextrin beverage had gastric fluid aspiration more than 20 ml, with *p* value of 0.014 as compared with only 2 non-obese patients. Out of these 6 patients, 2 patients were morbidly obese who were scheduled for OGDS assessment before bariatric surgery. About 40 cc residual gastric fluid was aspirated out in both patients. Similar with obese patient in group A, we found that 2 patients were morbidly obese have GRV more than 20 cc. However, no case of pulmonary aspiration was recorded. Thus, our findings can alert the physician or anesthetist to be more cautious with morbidly obese patients for longer fasting period prior to endoscopic procedure or general anesthesia. Obesity is associated with more difficult intubation and high incidence of gastroesophageal reflux disorder^22–24^. Therefore, obesity might be an independent anesthetic risk factor, regardless of the gastric emptying time. For this group of patients, the optimum protocol to deal with them needs further investigations.

A pre-operative prolonged fasting may result in patient's discomfort, thirst and weakness^25^, and a long fasting period may lead to glycemic imbalance and increase in catabolic mechanism due to starvation^26^, thus resulting in poor post-operative outcomes and complications. Currently, a shorter fasting period before surgical procedures requiring general anaesthesia has been tested with success. Practice guidelines developed by the American Society of Anesthesiologists (ASA) from their updated report in 2017 suggested for fasting from the ingestion of clear liquids for 2 or more hours before elective procedures requiring general anesthesia, regional anesthesia or procedural sedation and analgesia is safe and decreased risk of dehydration from prolonged fasting^4^. For gastroscopy, the European Society of Gastrointestinal Endoscopy (ESGE) guideline recommended that patient should be fasted at least 6 h of solid food and 2 h of clear fluid before the procedure^1^. This is to ensure safety and a clear endoscopic view. However, recent studies have refuted this recommendation when they demonstratedadequate endoscopic assessment with improved patient general comfort among those ingestion of clear fluid containing carbohydrate load 2 h before endoscopy^5, 6, 9^.

VAS was used to assess patient’s well-being in this study as similar to other study to measure different aspect of participants’ discomfort^17, 27^. Five variables were measured including hunger, thirst, anxiety, tiredness and general discomfort. In our study, both in carbohydrate loading and control group had a significant increase in participants’ well-being in all 5 parameters. The maltodextrin beverage group had significant improvement in patient’s well-being which was consistent with previous studies^5, 17, 28^. This result consistent with the initiation of carbohydrate loading to reduce pre-operative stress due to prolong starvation. The ingestion of carbohydrate loading of at least 2 h prior to operation or upper endoscopic procedure is safe, which is similar to other study^29^. Even though obese patients in maltodextrin beverage group had higher GRV, there was no aspiration noted in this population. There were no severe adverse events occurred during this study including pulmonary aspiration, failed or incomplete gastroscopy due to overt gastric residual volume.

The strength of this study is that patient was blinded on the type of fluids taken prior to gastroscopy as both is clear fluid and tasteless. This is also a randomised controlled trial and aspirated gastric fluid was measured by direct visualisation. Our study has some limitations as it is a single center study and small sample size numbers. In our study, the assessment of GRV via direct endoscopic visualisation were patient who indicated for gastroscopy and not for long hour surgery which may far from representing a preoperative situation. The outcome of OGDS is strictly related with visibility achieved during procedure. As Carborie® is a form of clear fluid, the impact towards visibility does not affect the mucosal assessment as described in POLPREP study^30^. Degree of anxiety may differ in patients prior to major operation as compared to before OGDS. Anxiety disturbs rapid fluid clearance from stomach and influence VAS score^31^. In the future, we recommend a randomised controlled trial with more sample size and co-morbidity to be included as the co-variates. More study is needed in patients with long standing DM and morbidly obese patients.

In our study, there is no significant difference in GRV and VAS well-being score for both groups. Therefore, in future practice, the patient undergoing OGDS would not need a strict fasting protocol. This data could also extrapolate in the use of maltodextrin as the pre-operative carbohydrate loading fluids prior to major surgery.

In conclusion, pre-endoscopy carbohydrate rich beverage is safe to be given 2 h prior to the gastroscopy. The GRV and VAS score showed significant improvement in the patient’s well-being; hunger, thirst, anxiety, tiredness and general discomfort in both groups before and after OGDS.

### Supplementary Information

Supplementary Tables.

## Data Availability

The datasets used or analysed during the current study are available from the corresponding author upon reasonable request.
