# Supplementary material for: The effect of pre-endoscopy maltodextrin beverage on gastric residual volume and patient’s well-being: a randomised controlled trial
Source: Sci Rep. 2023 Nov 16;13:20078. doi: 10.1038/s41598-023-47357-5 (PMC10654920; doi:10.1038/s41598-023-47357-5)
Supplement: Supplementary file 1 — Supplementary Tables. [file 41598_2023_47357_MOESM1_ESM.docx]

**Supplementary Table 1: Categorical gastric residual volume (GRV) of the two groups between patients with diabetes mellitus and without diabetes mellitus.**

|  | DM | Non-DM | *p* value* |
| --- | --- | --- | --- |
| Group A, n (%) |  |  |  |
| GRV ≤ 20 ml | 9 (69.2) | 21 (84.0) | 0.289 |
| GRV > 20 ml | 4 (30.8) | 4 (16.0) |  |
| Group B, n (%) |  |  |  |
| GRV ≤ 20 ml | 9 (81.8) | 23 (79.3) | 0.859 |
| GRV > 20 ml | 2 (18.2) | 6 (20.7) |  |
| **p* values were calculated using the Chi-square test.  Group A = patients were given 400 ml plain water (control group) 2 hours prior to the esophagogastroduodenoscopy (OGDS); Group B = patients were given 400 ml Carborie® (carbohydrate loading) 2 hours prior to the esophagogastroduodenoscopy (OGDS); GRV = gastric residual volume (i.e., categorical gastric residual volume with a cut-off of 20 ml was used based on scatter plots of gastric residual volume in both study groups which mostly showed the volume of below 20 ml); DM = diabetes mellitus. | | | |

**Supplementary Table 2: Categorical gastric residual volume (GRV) of the two groups between non-obese and obese patients.**

|  | Non-obese  BMI ≤ 27.5 kg/m^2^ | Obese  BMI > 27.5 kg/m^2^ | *p* value* |
| --- | --- | --- | --- |
| Group A, n (%) |  |  |  |
| GRV ≤ 20 ml | 16 (80.0) | 14 (77.8) | 0.867 |
| GRV > 20 ml | 4 (20.0) | 4 (22.2) |  |
| Group B, n (%) |  |  |  |
| GRV ≤ 20 ml | 23 (92.0) | 9 (60.0) | 0.014 |
| GRV > 20 ml | 2 (8.0) | 6 (40.0) |  |
| **p* values were calculated using the Chi-square test.  Group A = patients were given 400 ml plain water (control group) 2 hours prior to the esophagogastroduodenoscopy (OGDS); Group B = patients were given 400 ml Carborie® (carbohydrate loading) 2 hours prior to the esophagogastroduodenoscopy (OGDS); GRV = gastric residual volume (i.e., categorical gastric residual volume with a cut-off of 20 ml was used based on scatter plots of gastric residual volume in both study groups which mostly showed the volume of below 20 ml); BMI = body mass index. | | | |
